# The Feasibility and Safety of Laparoscopic Cholecystectomy Approach without the Intraopertative Cholangiography Use: A Retrospective Study on 750 Consecutive Patients

**DOI:** 10.4021/gr462e

**Published:** 2012-07-20

**Authors:** Kemal Atahan, Serhat Gur, Evren Durak, Atilla Cokmez, Ercument Tarcan

**Affiliations:** a Ataturk Educational and Research Hospital 1st Surgical Department, Izmir, Turkey

**Keywords:** Laparoscopic cholecystectomy, Intraoperative cholangiography, Gallstone disease

## Abstract

**Background:**

We have retrospectively reviewed the results of all common bile duct (CBD)-stone preoperative asymptomatic patients operated on our unit to point out the feasibility and safety of the laparoscopic cholecystectomy approach without the IOC use.

**Methods:**

From January 2004 and June 2008 we analyzed all the data from hospital records and follow up results of all the patients who underwent LC. The indications for performing preoperative endoscopic retrograde cholangiopancreatography (ERCP) or selective IOC were abnormal liver function tests, history of jaundice, cholangitis or pancreatitis, and ultrasonographic evidence of CBD stone or dilation (≥ 10 mm). These patients were excluded from study. The follow up of the all patients were done by liver function tests and abdominal ultrasonography when needed at the time of the visit.

**Results:**

Between January 2006 and June 2010, 750 patients were operated in our clinic. In 34 patients, operations were converted to open cholecystectomy (OC). Of these 750 patients, 98 of them had one or more exclusion criteria and were excluded from the further analyzes. We did not perform any IOC during LC. Regular follow up of at least two years was obtained in 618 (618/657, 94.0%) patients. No operative mortality was encountered among the patients. Postoperative morbidity was detected in 15 of the patients (2.5%). In one patient, CBD injury was detected (0.017%). The mean follow up was 35 (24 - 74) months. Retained stone was detected in three patients (3/577, 0.5%) during the follow up.

**Conclusion:**

This approach allows to omit routine IOC and to perform LC safely in selected patients group given the low percentage of both CBD injuries and symptomatic retained stones observed in the late follow up period in our 618 operated patients, we consider our approach a feasible and safe approach to manage patients with gallbladder stones re-confirming the results of other studies.

## Introduction

Laparoscopic cholecystectomy (LC) has been accepted as the gold standard for the treatment of gallstone disease worldwide. However there has been considerable controversy regarding the need for routine intraoperative cholangiography (IOC) for patients undergoing LC [1].

The advantages of IOC are to help to clarify anatomy and therefore reduce bile duct injuries during LC [2, 3]. In addition it detects asymptomatic bile duct stones which are thought to be up to 5% of patients undergoing LC [4-6]. Thus routine use of IOC lowers the morbidity associated with LC by reducing the risk of common bile duct (CBD) injuries, retained and asymptomatic stones [6]. Nevertheless, IOC has been shown to have a number of disadvantages. These are increased cost, prolonged operating time and false positive results leading to unnecessary CBD exploration [7, 8].

We have retrospectively reviewed the results of all CBD-stone preoperative asymptomatic patients operated on our unit to point out the feasibility and safety of the laparoscopic cholecystectomy approach without the IOC use.

## Material and Methods

In this retrospective study we gathered and analyzed all the data from hospital records, operative notes, cholangiographic studies, and follow up of all the patients who underwent LC between January 2006 and June 2010. Preoperative evaluations of the patients were done using abdominal ultrasonography, liver function tests and other routine laboratory tests. The indications for performing preoperative endoscopic retrograde cholangiopancreatography (ERCP) or selective IOC were abnormal liver function tests (ALP > 125 U/L, SGOT > 55 U/L, Bilirubin > 1.3 mg/dL), history of jaundice, cholangitis or pancreatitis, and ultrasonographic evidence of CBD stone or dilation (≥ 10 mm). These patients were excluded from the study. Also patients having gall bladder carcinoma or Mirizzi’s syndrome were excluded from the study. The exclusion criteria are shown in Table 1.

**Table 1:** The Exclusion Criteria

Patients who underwent preoperative ERCP or IC
Abnormal liver function tests
History of jaundice, cholangitis, pancreatitis
Common bile duct stone or dilatation (≥ 10 mm)
Patients having gall bladder carcinoma
Patients with Mirizzi’s syndrome
Patients for whom regular follow up was not obtained
Patients whose operations were converted to open cholecystectomy (except for the reason of CDB injury)

All the patients were recalled to hospital and questioned for the history of jaundice, pancreatitis or cholangitis after the LC. The follow up of the all patients were done by liver function tests and abdominal ultrasonography when needed at the time of this visit. The admission of patients to the hospital due to jaundice, cholangitis and biliary pancreatitis were recorded. The patients having any of the above complaints or positive findings for biliary stone during two years after LC were accepted as retained stone. The patients in whom regular follow up was not obtained were excluded from the study.

## Results

Between, January 2004 and June 2008, 750 patients were operated in our clinic. In 34 patients operations were converted to open cholecystectomy (OC). The conversion rate was 4.3% and the success rate was 95.7%. The causes of the conversion from LC to OC were severe adhesions in 30 patients, bleeding in 3 patients and CBD injury in one patient. Of these 750 patients 98 of them had one or more exclusion criteria and were excluded from further analysis. Regular follow up of at least two years was obtained in 618 (618/657, 94.0%) patients. We did not perform any IOC during LC. This patient population is selected as the study group. No operative mortality was encountered among the patients. Postoperative morbidity was detected in 15 of the patients (2.5%) and is shown in Table 2. In one patient CBD injury was detected (0.017%). This injury was recognized during the operation and the procedure was converted to open laparotomy. The common bile duct was repaired after T-tube insertion. T-tube cholangiography was normal and patient was discharged without any problems postoperatively 20th day. In the first year follow up of this patient, there was no problem. The mean follow up was 35 (24-74) months. In the follow up retained stone was detected in three patients **(**3/618, 0.5%). Of these 3 patients, two of them had acute biliary pancreatitis symptoms and one patient was detected with asymptomatic retained stone during routine follow up. Stones were successfully removed with ERCP. All the patients were discharged from the hospital uneventfully.

**Table 2:** The Morbidity of Patients

Wound infection	10
Wound haematoma	3
Incision hernia	1
CBD injury	1
Total	15

## Discussion

The use of IOC for the evaluation of choledocholithiasis has become more important especially in the era of LC. There are debates and lack of consensus in the routine or selective use of IOC [7, 9-11]. The main advantage for routine IOC may include factors such as the identification of unsuspected CBD stones as well as better definition of the extrahepatic ductal anatomy, which will help surgeons to avoid incidental injury to the bile duct [12, 13]. Nickkholgh A. et al [8] performed routine IOC to 1133 patients successfully during LC. In this group the incidence of CBD stones is 3.3% and the incidence of asymptomatic CBD stones is 1.2%. In the same study, the incidence of CBD stones in which selective IOC has been performed is 1.1% and CBD injury occurred in 0.09%of the patients. Wu SC et al [14] performed IOC during LC selectively to their 249 patients. They selected patients for IOC according to the history of clinical jaundice, elevated serum bilirubin, SGOT, ALP levels and over 9 mm CBD dilatation. In this group, the incidence of CBD stones is 25.6%. This rate is 30% in patients who had abnormal findings of above biochemical tests. They found out that the most sensitive finding is CBD dilatation and CBD stone rate is 53%. In another study, it’s shown that ALP, bilirubin, amylase and CBD dilatation at ultrasonography are independent predictive factors of CBD stones [15]. The reported incidence of false positive cholangiography rates vary from 2% to 16% [16, 17]. Varadarajulu et al [18] performed ERCP to 51 patients who had abnormal IOC findings during LC. The ERCP showed normal results in 18 patients (35.2%).

In our study, we performed preoperative ERCP according to the preoperative laboratory results and clinical findings. In the selected group where there is no suspicion of CBD stones, only 0.5% patients had retained stones and all of the patients were treated with ERCP successfully. In a similar study, Lepner U et al [19] showed that for at least 2 years of follow up period after surgery, retained CBD stones were verified in 1.5% of the patients. The incidence of retained CBD stones in selected patients is 0.43% in another study conducted by Nugent N et al’ s [20].

In 1990s, during the learning curve of LC, a higher rate of iatrogenic biliary tract injuries was reported [21]. Stewart and Way [22], in a review of patients who were referred to their tertiary center with iatrogenic biliary tract injuries identified two important reasons for CBD injury during LC; false identification of CBD as the cystic duct and aggressive efforts to stop bleeding. In this study, in selective IOC group CBD injury rate was 0.3% and routine IOC group CBD defect was 0%. Lepner U et al [19], in a study found no CBD injuries in 413 patients who did not undergo IOC. Amott D. et al [23] in a similar study, found one CBD injury in 155 patients. In a multi center study from Italy Nuzzo G. et al [24] reported 235 bile duct injuries, with an overall incidence of 0.42% in 56 591 patients. In our study CBD injury rate was found to be 0.017% in 577 patients, which is similar to above mentioned literature.

Lepner U et al [19], reported that retained CBD stones were verified in 1.5% patients during the follow up period, for at least 2 years after surgery. In a similar study with 23.3 months of follow up after surgery, retained CBD stones were found to be 0.43% [20]. In our study, the rate of retained CBD stones was 0.5%.

On the other hand Nickkholgh A. et al [8] compared the patients who had undergone selective or routine IOC. The sensitivity, specificity, negative predictive value and positive predictive values were 50%, 100%, 98.6% and 100% respectively in selectively IOC performed group. The same values were 97.4, 100, 99.8 and 100% in patients who underwent routine IOC. They concluded that routine IOC is a safe, accurate, quick and cost-effective method for the detection of bile duct anatomy and stones.

In a study by Lill et al [25] where LC was performed to 1022 patients, a total of five (0.5%) BDIs were detected. Symptomatic CBD stones were found in ten (0.9%) patients in follow-up. Authors conclude that in the current patient population, the routine use of IOC may not have reduced the incidence of BDIs and both the incidence of BDIs (0.5%) and symptomatic postoperative CBD stones (0.9%) remain low without the routine use of IOC. More importantly, Giger et al [26] used Swiss database to identify risk factors for BDI and to assess the effect of intraoperative cholangiography (IOC). Data of patients who underwent LC for acute or chronic cholecystitis between 1995 and 2005 in 114 different Swiss institutions were used in univariable and logistic regression analyses, 31,838 patients, were analyzed. The incidence of BDI was 0.3 per cent (101 patients), and did not change over time (P = 0.560). Comparison of groups with and without intraoperative cholangiography showed no difference in the incidence of BDI (both 0.3 per cent; P = 0.755) and BDIs missed during surgery (10 versus 8 per cent; P = 0.737). As a result, they concluded that male sex and prolonged laparoscopic surgery are independent risk factors for BDI during LC. Frequent use of IOC does not seem to reduce BDI or the number of injuries missed during surgery.

This study shows that routine IOC is not necessary for patients undergoing LC in a selected patient group which has no history of gallstone pancreatitis or jaundice, normal liver functions tests and a CBD diameter less than 10 mm. This approach allows to omit routine IOC and to perform LC safely in selected patients group given the low percentage of both CBD injuries and symptomatic retained stones observed in the late follow up period in our 618 operated patients, we consider our approach a feasible and safe approach to manage patients with gallbladder stones re-confirming the results of other studies
